# Accurate mobile malware detection and classification in the cloud

**DOI:** 10.1186/s40064-015-1356-1

**Published:** 2015-10-07

**Authors:** Xiaolei Wang, Yuexiang Yang, Yingzhi Zeng

**Affiliations:** College of Computer, National University of Defense Technology, Changsha, China; Information Center, National University of Defense Technology, Changsha, China

**Keywords:** Mobile malware detection, Android, CuckooDroid, Static analysis, Dynamic analysis, Classification, Signature detection, Anomaly detection, Mobile cloud service

## Abstract

As the dominator of the Smartphone operating system market, consequently android has attracted the attention of s malware authors and researcher alike. The number of types of android malware is increasing rapidly regardless of the considerable number of proposed malware analysis systems. In this paper, by taking advantages of low false-positive rate of misuse detection and the ability of anomaly detection to detect zero-day malware, we propose a novel hybrid detection system based on a new open-source framework CuckooDroid, which enables the use of Cuckoo Sandbox’s features to analyze Android malware through dynamic and static analysis. Our proposed system mainly consists of two parts: anomaly detection engine performing abnormal apps detection through dynamic analysis; signature detection engine performing known malware detection and classification with the combination of static and dynamic analysis. We evaluate our system using 5560 malware samples and 6000 benign samples. Experiments show that our anomaly detection engine with dynamic analysis is capable of detecting zero-day malware with a low false negative rate (1.16 %) and acceptable false positive rate (1.30 %); it is worth noting that our signature detection engine with hybrid analysis can accurately classify malware samples with an average positive rate 98.94 %. Considering the intensive computing resources required by the static and dynamic analysis, our proposed detection system should be deployed off-device, such as in the Cloud. The app store markets and the ordinary users can access our detection system for malware detection through cloud service.

## Background

As reported, Android is the most popular platform for Smartphone today with a market share of 84.7 % (Lindorfer et al. 2015). In contrast to other platforms, such as iOS which allow users to install apps only available in the iTunes App Store, Android allows to install applications from many sources, such as Google Play Store, third-party markets, torrents, or direct downloads, etc. Naturally, this liberty makes bundling and distributing applications with malware easy for attackers, who try to lure users into running malicious code, e.g. by repackaging popular apps with malicious payload (Lindorfer et al. 2015). Privacy breaches (e.g., access to address book and GPS coordinates), monetization through premium SMS and calls, other harmful malicious attack (Shang et al. 2011) have become real threats. Although there have already been some drive-by download sightings for Android (Protalinski 2012), the most prevalent infection vector is still user-based installation.

Several security measures have been proposed by the Android platform providers to prevent the installation of malware, most notable of which is the Android permission system. Each application has to explicitly request some permission from the user during the installation to perform certain tasks on the device, such as sending SMS message, etc. (Arp et al. 2014). However, many users tend to blindly grant permission to unknown applications and thereby undermine the purpose of the permission system. To help users, some information sources (Lindorfer et al. 2015) are provided for them to decide whether or not to install an app, such as trustworthiness of the app’s origin, app reviews by other users, results from antivirus (AV) scanners, results from Google’s app verification service, etc. However, as introduced in Lindorfer et al. (2015), all of these sources have major shortcomings and cannot prevent the installation of malware efficiently.

To solve this problem, many research methods have been proposed for analyzing and detecting Android malware prior to the installation. These methods are mainly categorized into two generic approaches, namely static analysis and dynamic analysis. For example, TaintDroid (Enck et al. 2010), DroidRanger (Zhou et al. 2012) and DroidScope (Yan and Yin 2012) are dynamic analysis methods that can monitor the behavior of applications during runtime. Although very effective in identifying malicious activity, run-time monitoring suffers from significant overhead and cannot be directly applied on mobile devices. In addition, pure dynamic analysis systems are prone to analysis evasion. By contrast, static analysis methods, such as Drebin (Arp et al. 2014), RiskRanker (Grace et al. 2012), introduce only small run-time overhead, but struggle with increasingly popular obfuscation and dynamic code loading techniques.

In this paper, we proposed a hybrid mobile malware detection and classification system by extending a new open source analysis framework CuckooDroid (CuckooDroid 2015) to detect and classify malware accurately before installation. Our proposed system is designed for both app markets and ordinary users. For app markets, our system can perform a large-scale detection and classification aided by an automated and comprehensive analysis with CuckooDroid. For ordinary users, this detection and classification system can be provided as a service through mobile cloud service (MCS). In addition, a detailed report that is easy to grab and understand is provided, which is generated by CuckooDroid. Our proposed detection system mainly consists of two parts: anomaly detection engine and signature detection engine. Firstly, by using dynamic analysis results, anomaly detection engine can detect new zero-day and unknown malware, as done in Sahs and Khan (2012). During the dynamic analysis, some vital dynamic features of an app in runtime are tested in runtime during dynamic analysis, such as SMS, Phone, dynamic code loading, etc. The anomaly detection engine is built on one-class support vector machine classifiers. Secondly, the signature detection engine which is built based on linearSVC classifier is responsible for detecting and classifying known malware or new variants using static and dynamic analysis results. During the static analysis, many features from the source code and manifest are extracted as possible, as did in Arp et al. (2014). Aided by the static and dynamic analysis results, signature detection engine can efficiently detect new variants and identify their corresponding families through classification.

Note that the features collected during static and dynamic analysis are organized in sets of strings (such as permissions, receivers, hardware) and embedded in a joint vector space. Then each application is represented with a feature vector which can be fed to a certain machine learning technique. Due to the intensive computing resources required by the static and dynamic analysis, both anomaly detection engine and signature detection engine should be deployed off-device, such as in the Cloud. Using a classifier that is trained on a large set of known malicious apps (malware) and benign apps (goodware), our proposed system can detect whether a new app is abnormal or not firstly. Once a new app is detected as abnormal by anomaly detection engine, it is malware sample with high probability in our system. Therefore, a further comprehensive analysis by signature detection engine is started to analyze which family this malware belongs to. In case a new app is detected as normal by anomaly detection engine, we assume it is benign in this paper. The assumption is reasonable according to the high true detection rate of anomaly detection engine in experiments, which we will discuss it in detail in evaluation part.

In summary, our contributions are as follows:

1. *Effective malware detection and classification* Based on two phase detection by static analysis and dynamic analysis respectively, our proposed system is capable of detecting and classifying malware with high accuracy and few false alarms.
2. *Zero*-*day malware and new variants detection* Our proposed hybrid detection system consists of two phase: anomaly detection engine and signature detection engine. Anomaly detection engine is coarse-grained and can detect new malware which is anomalous from a large number of benign apps. Signature detection engine is a fine-grained, which can detect known malware or new variants of a known family. Experiment results show that the two detection engines both achieve high true positive accuracy and low false negative.
3. *Integrating anomaly detection and misuse detection* Considering the fact that the purely anomaly detection has a relative high false positive rate and the purely misuse detection has a relative high false negative rate, we integrate them to achieve high true positive and low false negative. As we know, we are the first to do this in mobile malware detection.
4. *Detailed analysis reports* Our proposed system generates a detailed analysis report that is easy to understand during the detection, which includes the extracted static and dynamic information.
5. *System implementation* We implemented our proposed detection system using CuckooDroid. Based on this implementation, many experiments are executed to evaluate the performance of this system.

The rest of this paper is organized as follows: related work is introduced in “Related work”. Architecture overview is presented in “Architecture overview”. Our proposed system implementation and evaluation are discussed in detail in “Implementation” and “Evaluation”, respectively. “Discussion” concludes the paper.

## Related work

In the last years, mobile malware detection has been a hot area of research, especially android malware detection. To counter the growing amount and sophistication of this malware, a large number of concepts and techniques have been proposed and are mainly categorized to: (1) static analysis; (2) dynamic analysis. A detailed and comprehensive review of the current mobile malware detection is provided in the studies of Zhou and Jiang (2012) (Suarez-Tangil et al. 2013; Sufatrio et al. 2015; Faruki et al. 2015). And since that we use the machine learning in our detection system, the related work of machine learning based detection is introduced.

### Detection using static analysis and limitation

The first approaches for detecting Android malware have been inspired by concepts from static program analysis. A static analyzer inspects an app by just disassembly, de-compilation without actually running it, hence does not infect the device. Since it analyzes an app’s whole source or recovered code, the analyzer can achieve high code coverage.

A large number of methods that inspect applications and disassemble their code have been proposed (e.g. Arp et al. 2014; Lindorfer et al. 2015; Grace et al. 2012; Aafer et al. 2013; Chakranomaly et al. 2013; Chin et al. 2011; Zhu et al. 2014. RiskRanker (Grace et al. 2012) detects high and medium risk apps according to several predetermined features, such as the presence of native code, the use of functionality that can cost the user money without her interaction, the dynamic loading of code that is stored encrypted in the app, etc. Comdroid (Chin et al. 2011) analyze the vulnerability in inter-app communication in Android apps and find a number of exploitable vulnerabilities. DroidAPIMiner (Aafer et al. 2013) and Drebin (Arp et al. 2014) classify apps based on features learned from a number of benign and malicious apps during static analysis. An app recommender system is proposed in Zhu et al. (2014) to rank apps based on their popularity as well as their security risk, considering requested permissions only. FlowDroid (Arzt et al. 2014) performs a flow-, context-, object-, and field-sensitive static taint analysis on Android apps. It models Android app’s lifecycle states and handles taint propagation due to callbacks and UI objects. As the most closely related to our signature detection engine module, some static features such as permissions, intent filters, and the presence of native code are also extracted in MAST (Chakranomaly et al. 2013) to perform market-scale triage and to select potentially malicious samples for further analysis.

#### The limitation of static analysis

Static analysis lacks the actual execution path and relevant execution context. Moreover, there exist challenges in the presence of code obfuscation as well as dynamic code loading (Poeplau et al. 2014). All those approaches lack the ability to analyze code that is obfuscated or loaded dynamically at runtime, a prevalent feature of apps as evidenced by a recent large scale study (Lindorfer et al. 2014), unless they are complemented by some form of dynamic analysis, as recently proposed in StaDynA (Zhauniarovich et al. 2015).

#### Our solution to the limitation of static analysis

In contrast, our proposed system does not suffer from those limitations, since our anomaly detection engine performs abnormal detection firstly through dynamic analysis.

### Detection using dynamic analysis and limitation

Static analysis and detection approaches are quick, they fail against the encrypted, polymorphic and code transformed malware. In order to overcome the shortcomings of static analysis, some dynamic analysis based methods (Zhang et al. 2013b; Yan and Yin 2012; Enck et al. 2010; Burguera et al. 2011; Wu and Hung 2014; Gilbert et al. 2011; Rastogi et al. 2013) are proposed. Dynamic analysis is conducted by executing an app, on either a real or virtual execution environment such as the Android Virtual Device (AVD), and observing the app during its execution.

The analysis system TaintDroid (Enck et al. 2010) and DroidScope (Yan and Yin 2012) are the most notably, which enable dynamically monitoring applications in a protected environment. TaintDroid focuses on taint analysis and DroidScope make introspection at different layers of the platform. Although both systems provide detailed information about the behavior of apps, they require too many resources to deploy on Smartphones directly.

A first step towards the use of dynamic analysis results for Android malware detection is anomaly detection engine by CrowDroid (Burguera et al. 2011), which performs k-means cluster based on system-call counts. The number of invocations of API and system calls is selected as coarse-grained features to train various classifiers to analyze apps. However, their monitoring approach relies on modifying the app under analysis, which can be easily detected by malware. Another related approach combining static with dynamic analysis is DroidDolphin (Wu and Hung 2014). Again, the approach relies on repackaging and injecting an app with monitoring code. Although the authors observed that the accuracy increased with the size of the training set, DroidDolphin (Wu and Hung 2014) achieves an accuracy of only 86.1 % in the best case. At the meantime, these dynamic analysis methods are all prone to analysis evasion due to the increasing use of emulator detection technology in malware. VetDroid is a dynamic analysis platform for reconstructing sensitive behaviors in Android apps from a permissions use perspective (Zhang et al. 2013b). Zhang et al. points out that traditional system call analysis is not appropriate for characterizing the behaviors of Android apps, as it misses high-level Android-specific semantics and fails at reconstructing IPC and RPC interactions. Afonso et al. (2014) dynamically analyze Android apps using the number of invocations of API and system calls as coarse-grained features to train various classifiers. However, their monitoring approach relies on modifying the app under analysis, which is easily detectable by malware. AppsPlayground (Rastogi et al. 2013) performs a TaintDroid-based dynamic taint tracing, API monitoring, and kernel-level monitoring. Event triggering and intelligent execution techniques are adopted to realize comprehensive execution coverage and achieve code coverage of 33 %.

#### The limitation of dynamic analysis

Although dynamic analysis surpasses the static analysis in many aspects, dynamic analysis also has some drawbacks. Firstly, dynamic analysis requires too many resources relative to static analysis, which hinders it from being deploying on resource constraint smartphone. Secondly, dynamic analysis is subject to low code coverage. Sasnauskas and Regehr (2014) mentioned that producing highly structured inputs that get high code coverage is an open research challenge. Thirdly, recently malware attempts to detect the emulator and other dynamic analysis systems (Vidas and Christin 2014; Petsas et al. 2014; Jing et al. 2014), avoiding launching their payloads. Thus, some dynamic analysis systems are prone to analysis evasion.

#### Our solution to the limitation of dynamic analysis

On contrast to the above mentioned methods, anomaly detection engine in our proposed detection system performs dynamic analysis through Dalvik Hooking based on Xposed Framework. Therefore, our analysis module is difficult to be detected by avoiding repackaging and injecting monitoring code. As we know, most of dynamic analysis methods don not integrate the anti-emulator tools and thus are prone to analysis evasion. To solve this problem, some emulator anti-detection tools (such as Content Generator, etc.) are integrated to make a more transparent dynamic analysis environment, which can avoid emulator detection at a certain extent and extract more valuable dynamic information. As for code coverage, we adopt MonkeyRunner (Android Developers 2015) to stimulate the inputs during app execution.

### Detection using machine learning and limitation

The difficulty of manually crafting and updating detection patterns for Android malware has motivated the application of machine learning. Several methods have been proposed to detect and analyze applications automatically using machine learning methods (e. g. Arp et al. 2014; Lindorfer et al. 2015; Grace et al. 2012; Aafer et al. 2013; Afonso et al. 2014; Spreitzenbarth et al. 2013; Amos et al. 2013). For example, the method proposed in Arp et al. (2014) applies linearSVC learning methods to the static features of applications for detecting malware. Similarly, the methods RiskRanker (Grace et al. 2012) and DroidAPIMiner (Aafer et al. 2013) use machine learning techniques to detect malware with features statically extracted from Android applications. In contrast, the method proposed in Afonso et al. (2014) detects malware with a machine learning technique and dynamically extract features. A framework is proposed in Amos et al. (2013) to evaluate mobile malware classifiers based on the same features as Andromaly with an equally limited testing set of only 50 applications. Additionally, the tested classifiers achieve substantial false positive rates ranging from 14.55 % up to 44.36 %, rendering them completely impractical. Closest to our work are MARVIN (Lindorfer et al. 2015) and MobileSandbox (Spreitzenbarth et al. 2013), which use the static and dynamic features by machine learning and achieve high accuracy.

#### The limitation of machine learning based detection

Overall, previous work focuses on detecting malware using machine learning techniques, which are either misuse-based detection or anomaly-based detection. Misuse based detector tries to detect malware based on signatures of known malware. Misuse detector is specifically designed to detect known malware, leading to low number of false alarms. However, misuse detector could not detect zero-day malware. Anomaly detector refers to identifying malware that is anomalous with respect to the normal apps. Despite their capability in detecting zero-day malware, anomaly detector suffers from high false positive rate. The misuse and anomaly detector are complementary.

#### Our solution to the limitation of machine learning based detection

Hence, by taking advantages of low false-positive rate of misuse detector and the ability of anomaly detector to detect zero-day malware, a hybrid malware detection method is proposed in this paper, which is the novelty in this paper.

## Architecture overview

**Fig. 1:**
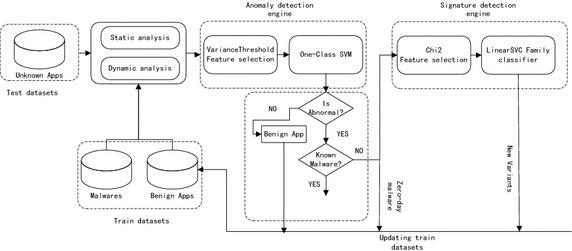
System overview

As described in Fig. 1, our proposed detection system mainly consists of two engines: anomaly detection engine and signature detection engine. Anomaly detection engine is responsible for performing zero-day malware detection through dynamic analysis. And signature detection engine is responsible for performing new variant detection by combining static analysis results with dynamic analysis results. Signature detection engine is trained on known malware and benign apps.

Considering the resource-consumption process of detection and the constraint computing resource on mobile devices, both anomaly detection engine and signature detection engine should be deployed off-device at somewhere with rich resources, such as in the Cloud.

The process is outlined as follows:

### Static and dynamic analysis

**Fig. 2:**
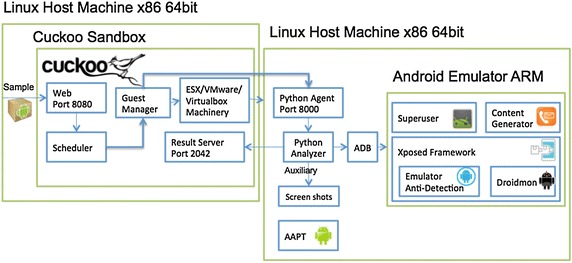
Framework of Cuckoodroid

Firstly, all the train datasets and test datasets are processed statically and dynamically. As done in Drebin (Arp et al. 2014), we extract the static features from the manifest file and the disassemble dex code. In order to extract the dynamic features of apps during runtime, CuckooDroid is used to run the apps in an emulator environment. As shown in Fig. 2, CuckooDroid is composed of one manage node, and a number of slave nodes, which can be either Android emulators or linux-based virtual machines in the Cloud. Contrast to other dynamic analysis, CuckooDroid has integrated a collection of known emulator anti-detection techniques for hiding the Android emulator and providing a transparent analysis environment. At the meantime, a Dalvik API hooking based on Xposed framework is adopted to capture the dynamic API calls and information. Also the analysis results of submitted app are stored in a database in our proposed method. Through this way, when a submitted app has been analyzed before, its analysis results will be returned directly.

### Anomaly detection

Anomaly detection engine is responsible for detecting normal and abnormal apps through dynamic analysis and providing a preliminary analysis results. In contrast to signature detection, dynamic features will be used in anomaly detection and be embedded into vector space. Also a Variance treshold- based feature selection method is applied to these feature vectors. In order to detect abnormal apps, a One-Class SVM classifier model is built on benign apps. A new app will be labeled as either zero-day malware or benign app by this trained classifier. When an unknown app is submitted, its feature vector will be fed to the classifier and a decision about whether is malware or not is made.

If an unknown app is categorized as abnormal and it is not known malware, further signature detection will be triggered to classify this malware and determine which family it belongs to.

In order to maintain the detection accuracy of the two detection engines, all the new variants, zero-day malware and benign apps will be stored to update the training dataset at a specific period.

### Signature detection

At first, the extracted static and dynamic string features will be embedded into vector space, generating feature vectors. Then, a Chi^2^-based feature selection method is applied to these feature vectors.

During signature detection, the feature vectors of malicious and benign apps will be generated first, as stated above. Then a linearSVC classifier model is trained based on these train feature vectors which consist of known malware and benign apps.

The detected abnormal app during anomaly detection will be further classified using a multi-family classifier. When the classification ends, the detected abnormal app will be classified into a certain malware family. Since the detected malware is unknown, it will be a new variant of a family with a high probability.

## Implementation

### Feature extraction

Feature extraction is an essential part of both anomaly detection engine and signature detection engine. Both the static analysis and dynamic analysis are performed before anomaly detection and signature detection. CuckooDroid is used to extract the dynamic features of each app. At the meantime, some static features are extracted.

#### Static analysis features

For android apps, static analysis can provide a rich feature set about the app, such as requested permissions, registered activities, etc. In this paper, our static analysis mainly focus on the manifest and the disassembled dex code of the app, which both can be obtained by a linear sweep over the app’s source code and files. We adopt the Android Asset Packaging Tool to extract the static features, as Drebin (Arp et al. 2014). Additionally, several aspects of the app’s code are statically determined in case they might not be triggered during the dynamic analysis phase, as done in (Lindorfer et al. 2015), such as the use of reflection API, the dynamic loading of code, the use of cryptographic API. Specially, the static feature extracted mainly includes two parts: the static features from manifest and disassembled code.

Every application developed for Android must include a manifest file, which provides data supporting the installation and later execution of the application. As did in Arp et al. (2014), we extract the information stored in this file. The specific static features extracted include: Hardware components, Activities, Intents-filters, etc. Also, some static signatures about an app are generated according to the extracted static information, such as “Application request dangerous permission”, “Application uses native code”, etc. Compared to Arp et al. (2014), we do not extract the network address features and restricted API calls features. The detailed extracted static features are shown in Table 1.

**Table 1:** Categories and numbers of extracted features

Source	Category	#Feature
Dynamic	File operations	19,038
Static and dynamic	Signatures	1283
Dynamic	Registered_receivers	8334
Dynamic	Reflection_calls	14,799
Static	Used/required permissions	1267
Static and dynamic	SMS, phone, contacts	1493
Static	Application components	21,523
Static and dynamic	Dynamic code loading	916
Static and dynamic	Crypto operation	41
Dynamic	Data_leak	828
Dynamic	Commands	937
Static and dynamic	Network activity	37,734
Static	The use of special API	20,162
Dynamic	System properties	13,081

#### Dynamic analysis features

As research on ×86 malware detection, purely static analysis techniques are prone to evasion by some anti-detection techniques, such as code obfuscation, etc. In order to prevent attackers from evading the learning method, e.g. with mimicry attacks (Šrndić and Laskov 2014), features should inherently represent the malicious behavior to be detected. Thus the corresponding dynamic analysis features capturing the harmful behavior should be extracted.

In order to extract the dynamic analysis features, we extend the open-source and automated dynamic analysis framework CuckooDroid proposed in CuckooDroid (2015). CuckooDroid performs dynamic analysis at Dalvik-level through a Dalvik API monitoring based on Xposed framework. In addition, a new stimulation tool is integrated to trigger program behavior and increase code coverage Robotium (Robotium 2014), which is used to enhance original CuckooDroid by simulating user’s interactions with the mobile apps and can automate the testing process. During the dynamic analysis, we monitor the following events: “File access and operations”, “Register receivers”, “Executed commands”, “Content resolver queries”, “Telephony Manager listen”, “Find resource”, “Dynamic suspicious calls”, “SMS”, “Phone Events”, “Data leaks”, “Network operations”, etc. Compared to (Lindorfer et al. 2015), we also extract the crypto keys that apps use, then the encrypted traffic can be transformed to plaintext traffic.

### Embedding into vector space

The extracted static analysis features and dynamic analysis features are expressed as strings, which cannot be fed to machine learning directly. For example, a malware sample sending premium SMS messages may contain the requested permissions “SEND_SMS”, and the hardware components “android.hardware.telephony”. During our evaluation on dataset of 11,560 benign and malware samples, we extracted 190,367 different static and dynamic features, as shown in Table 1.

As most machine learning methods operate on numerical vectors, we need to map the extracted feature sets into a vector space first. Thus, we need to represent an app as an appropriate vector in order to machine learning algorithm. To this end, we use a simple bag of words representation. Firstly, we defines a features set S, which comprises all observable extracted string features. Secondly, a |S|-dimensional vector space can be defined using the feature set S, where each dimension is either 0 or 1. Then an app X can be mapped to this space by constructing a vector $\upvarphi ({\text{X}})$, for each feature s extracted from X the corresponding dimension is set to 1 and all other dimensions are set to 0.

$$ \upvarphi :{\text{X }} \to \{ 0, 1\}^{{|{\text{s}}|}} ,\quad \upvarphi \left( {\text{x}} \right) \to \left( {{\text{I}}\left( {{\text{x}},{\text{s}}} \right)} \right)_{{{\text{s}} \in {\text{S}}}} , $$

where the indicator function ${\text{I}}({\text{x}},{\text{s}})$ is simply defined as

$$ {\text{I}}\left( {{\text{x}},{\text{s}}} \right) = \left\{ \begin{aligned} &1 \quad {\text{if the application x contain features }} \hfill \\ &0 \quad {\text{otherwise}} \hfill \\ \end{aligned} \right. $$

### Choosing a classifier

When got feature vectors of apps, our proposed hybrid system uses different classifiers to perform anomaly detection and signature classification. In this paper, 1-class SVM algorithms and linear classifier are used to build the anomaly detection models and signature classification models, as introduced below.

#### Anomaly detection

Consider a data set of n observations from the same distribution described by p features. Consider now that we add one more observation to that data set. Is the new observation so different from the others that we can doubt it is regular? (i.e. does it come from the same distribution?) Or on the contrary, is it so similar to the other that we cannot distinguish it from the original observation? This is the question addressed by the novelty detection tools and methods.

In general, it is about to learn a rough, close frontier delimiting the contour of the initial observations distribution, plotted in embedding ***p***-dimensional space. Then, if further observations lay within the frontier-delimited subspace, they are considered as coming from the same population than the initial observations. Otherwise, if they lay outside the frontier, we can say that they are abnormal with a given confidence in our assessment.

A one-class support vector machine (1-class SVM) is a popular anomaly detection algorithm in various applications. In our proposed detection system, we use the novelty detection provided by 1-class SVM in Scikit-learn to detect anomaly malware.

The One-Class SVM has been introduced by Schölkopf et al. for that purpose and implemented in the Support Vector Machines module in the *svm. OneClassSVM* (http://www.scikitlearn.org/stable/modules/generated/sklearn.svm.OneClassSVM.html#sklearn.svm.OneClassSVM) object. It requires the choice of a kernel and a scalar parameter to define a frontier. The RBF kernel is usually chosen, although there exists no exact formula or algorithm to set its bandwidth parameter. This is the default in the scikit-learn implementation, we also choose the RBF kernel. The ‘nu’ parameter (we set nu = 0.01), also known as the margin of the One-Class SVM, corresponds to the probability of finding a new, but regular, observation outside the frontier. The ‘gamma’ parameter is set to 0.01, too.

#### Signature detection

The signature detection is responsible for classifying abnormal malware to its family. Due to that we classify the abnormal malware by the static and dynamic features, thus, the chosen classifier should adapt to high-dimensional feature space, as we extract 190,367 different static and dynamic features. Also the chosen classifier should adapt to sparse data, considering the single app only exhibit a small subset of the possible features.

Given these characteristics, we explore three machine learning approaches: a linear SVC classifier with L1 regularized logistic regression, a linear SVC classifier with L2 regularized logistic regression and a Support Vector Machine classifier (LinearSVM). Here, we apply *sklearn.svm* (http://www.scikitlearn.org/stable/modules/generated/sklearn.svm.OneClassSVM.html#sklearn.svm.OneClassSVM) to perform these three classifiers.

#### LinearSVC classifier

Compared to SVM, linear SVC implements “one-vs-the-rest” multi-class strategy. Given a feature vector $\upvarphi$ (*x*↽, a linear classifier computes the scalar product with a weight vector $\vec{w}:y = \mathop \sum \nolimits_{i} x_{i} w_{i}$. The outcome, *y*, is the margin of the classification.

Similar to SVC with parameter kernel=’linear’, linear SVC implemented in terms of liblinear rather than libsvm, so it has more flexibility in the choice of penalties and loss functions and should scale better (to large numbers of samples). Furthermore, linear SVC supports both dense and sparse input and the multiclass support is handled according to a one-vs-the-rest scheme.

As suggested in (Lindorfer et al. 2015), linear SVC with L1 regularization are superior to linear SVC with L2 regularization when dealing with many irrelevant features, while linear SVC with L2 regularization is extremely sensitive to the presence of irrelevant features. We show in our evaluation in Sect. 5 that both methods lead to similar results during classification, while linear SVC with L2 classifier performs slight better.

#### LinearSVM classifier

In principle, an SVM works the same way as a linear classifier. However, it does address one problem of linear classifiers: as the name already suggests, the latter classifies samples only accurately if the problem is linearly separable. To overcome this limitation, SVMs use the “kernel trick”, implicitly mapping the input into an even higher-dimensional space, where the problem is more easily separable.

However, the LinearSVM implementation is based on libsvm. The fit time complexity is more than quadratic with the number of samples which makes it hard to scale to dataset with more than a couple of 10,000 samples.

Furthermore, as further detailed in “Evaluation”, pure linear classification performs better than LinearSVM and has high classification accuracy. Thus, our signature classification chooses purely linear classifier.

### Feature selection

The features extracted during feature extracted include a large number of static and dynamic features, as we introduced in Table 1. However, this not means that they are all useful for anomaly detection and signature classification. To improve the performance and accuracy of our hybrid detection system, we apply different feature selection methods to anomaly detection and signature classification.

The classes in the *sklearn.feature_selection* (http://www.scikitlearn.org/stable/modules/generated/sklearn.svm.OneClassSVM.html#sklearn.svm.OneClassSVM) module can be used for feature selection/dimensionality reduction on sample sets, either to improve estimators’ accuracy scores or to boost their performance on very high-dimensional datasets. So we choose this class to perform our feature selection.

#### For anomaly detection

*VarianceThreshold* is a simple baseline approach to feature selection. It removes all features whose variance doesn’t meet some threshold. By default, it removes all zero-variance features, i.e. features that have the same value in all samples. In our system, the parameter of *VarianceThreshold* is set “threshold = (0.12 × (1 − 0.12))”.

#### For signature classification

Univariate feature selection works by selecting the best features based on univariate statistical tests. It can be seen as a preprocessing step to an estimator. Scikit-learn exposes feature selection routines as objects that implement mutiple transform methods, includes: *SelectKBest()*, *SelectPercentile()*, *Generic UnivariateSelect()*. In our system, we implement the *SelectKBest* method to removes all but the k highest scoring features. The parameter of *SelectKBest* is set “score_func = chi2, k = 2000”.

## Evaluation

After presenting our hybrid detection system in detail, we now proceed to an empirical evaluation of its efficacy.

### Dataset

For all experiments, we consider a dataset of real Android applications and real malware. To acquire benign apps, we design our crawler and craw a large number of apps in China app stores, such as http://www.appchina.com, http://www.as.baidu.com, http://www.mm.10086.cn, etc. To collect benign apps, we submit the crawled apps to VirusTotal, we label an app as benign if it does not trigger a response from 55 AV scanners used by VirusTotal. The malware samples we used in experiment are acquired from Drebin (Arp et al. 2014), The malware samples have been collected in the period of August 2010 to October 2012 and were anomaly detection engine available to us by the MobileSandbox project (Spreitzenbarth et al. 2013). The final dataset contains 6000 benign apps and 5560 malware samples. As clamed in Arp et al. (2014), this is one of the largest malware datasets that has been used to evaluate a malware detection method on Android.

An overview of the top malware families in our dataset is provides in Table 2, which contains several families that are currently actively distributed in app markets.

**Table 2:** Top 20 malware families in our dataset

Id	Family	#	Id	Family	**#**
A	FakeInstaller	925	K	Adrd	91
B	DroidKungFu	667	L	Droiddreru	81
C	Plankton	625	M	LinuxLotoor	70
D	Opfake	613	N	GoldDream	69
E	GingerMaster	339	O	MobileTx	69
F	BaseBridge	330	P	FakeRun	61
G	Iconosys	152	Q	SendPay	59
H	Kmin	147	R	Gappusin	58
I	FakeDoc	132	S	Imlog	43
J	Geinimi	92	T	SMSreg	41

### Anomaly detection performance

In this experiment, we evaluate the detection performance of our anomaly detection engine and compared it with other related detection approaches, some common AV scanners.

#### Detection performance of anomaly detection engine

Firstly, a one-class SVM model is trained using 4000 benign samples. Then we test our 5560 malware samples on this model. The false negative rate is 1.16 %, which indicates that 65 malware samples are not detected.

Next, we used the remaining 2000 benign samples as test samples to evaluate the false positive rate of our anomaly detector. The result shows that 1.30 % of benign apps are mistakenly recognized as abnormal apps during anomaly detection. This means, if our anomaly detector is applied to Google Play, a world’s android app market, among the approximately 1200 new apps per day, around 15 apps will be mislabeled as abnormal. This anomaly detection accuracy has surpassed the proposed methods in Zhang et al. (2014).

**Fig. 3:**
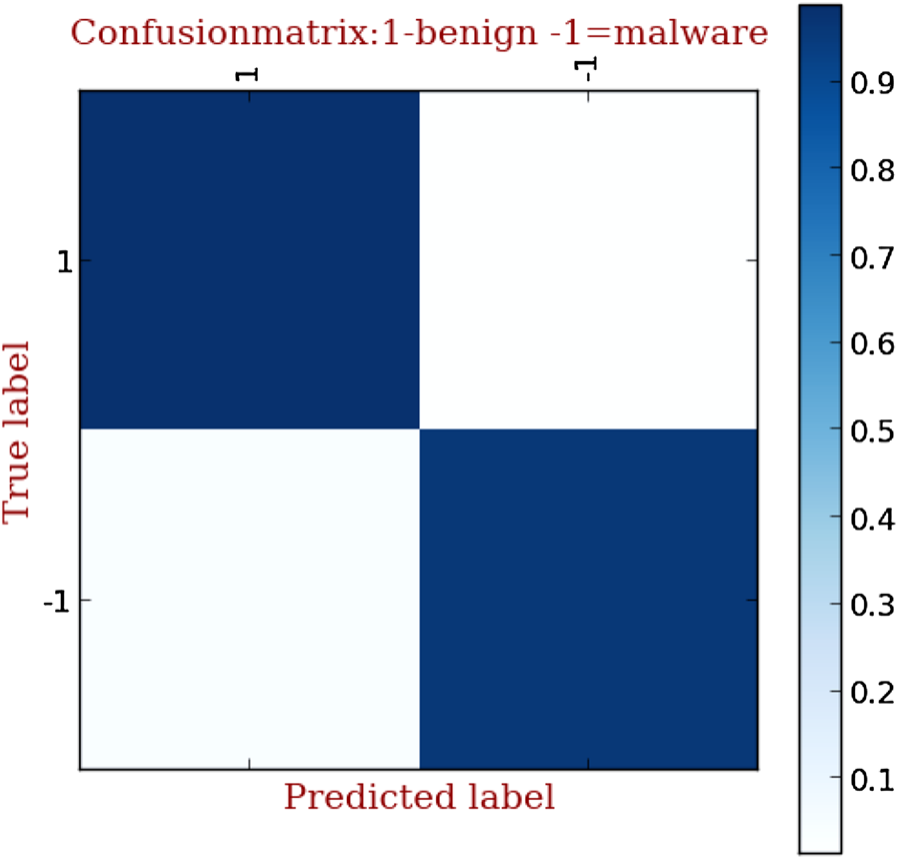
The confusion matrix of anomaly detection

Here we use a confusion matrix to characterize the performance of anomaly detection engine, as shown in Fig. 3. From the confusion matrix, it is obvious that our anomaly detection engine can accurately detect abnormal malware.

#### Compared it with other related detection approaches

**Fig. 4:**
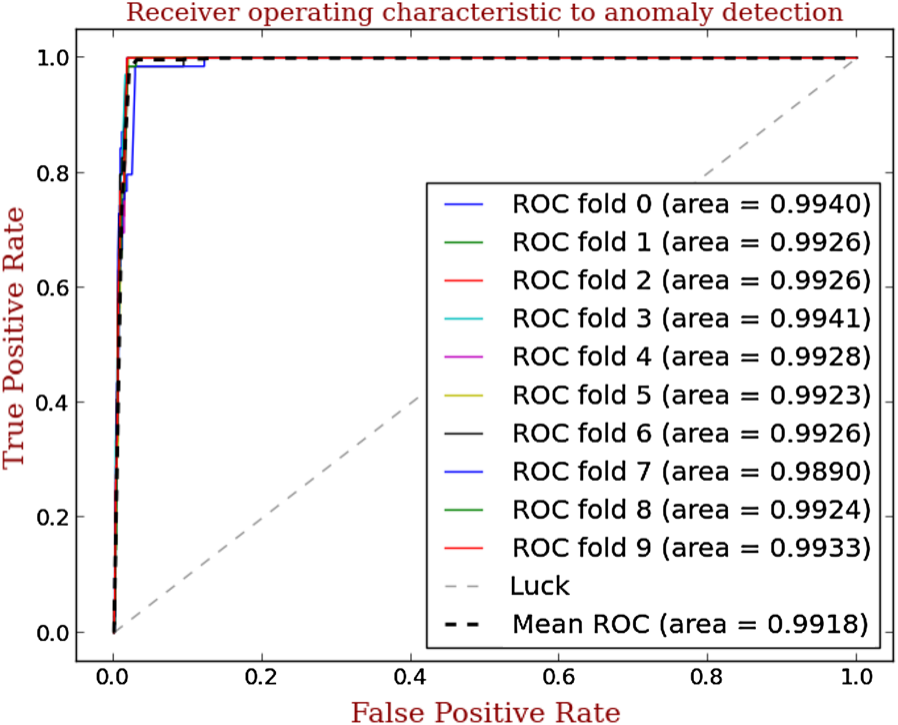
The ROC curve of anomaly detection

As discussed above, our anomaly detection engine anomaly detection engine can detect malware samples with true positive rate of 98.84 %, false negative rate of 1.16 %. Also anomaly detection engine can correctly label benign apps with a true positive rate of 98.7 %, a false negative rate of 1.3 %. To evaluate the performance accurately, a 10-fold cross validation is further performed, which is shown as ROC curve in Fig. 4.

**Fig. 5:**
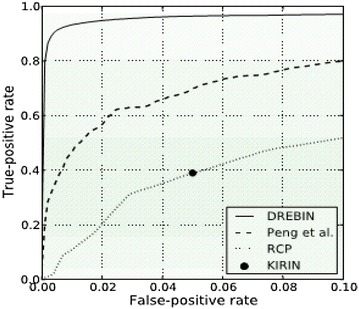
The ROC curve of other detection methods

As a comparison, we use the ROC figures in (Arp et al. 2014), as shown in Fig. 5. It is obvious that our anomaly detection engine outperforms other related detection methods.

Due to that our anomaly detection engine uses benign apps to train model, thus, no malware sample of certain families are required in advance. Therefore, whether our anomaly detection engine can detect unknown families are worth studying. To evaluate the detection unknown families malware performance of our anomaly detection engine, we use the top 20 malware families as test dataset. The detection rate of each family based on our anomaly detection engine is shown in Table 3, with an amazing result. All families except for FakeInstaller can be detected with a true rate of 100 %. Two instances of FakeInstaller are falsely labeled as benign apps.

**Table 3:** The detection rate of top 20 malware families

Family	Detection rate (%)	Family	Detection rate (%)
A	99.71	K	100
B	100	L	100
C	100	M	100
D	100	N	100
E	100	O	100
F	100	P	100
G	100	Q	100
H	100	R	100
I	100	S	100
J	100	T	100

#### Compared it with other AV scanners

**Fig. 6:**
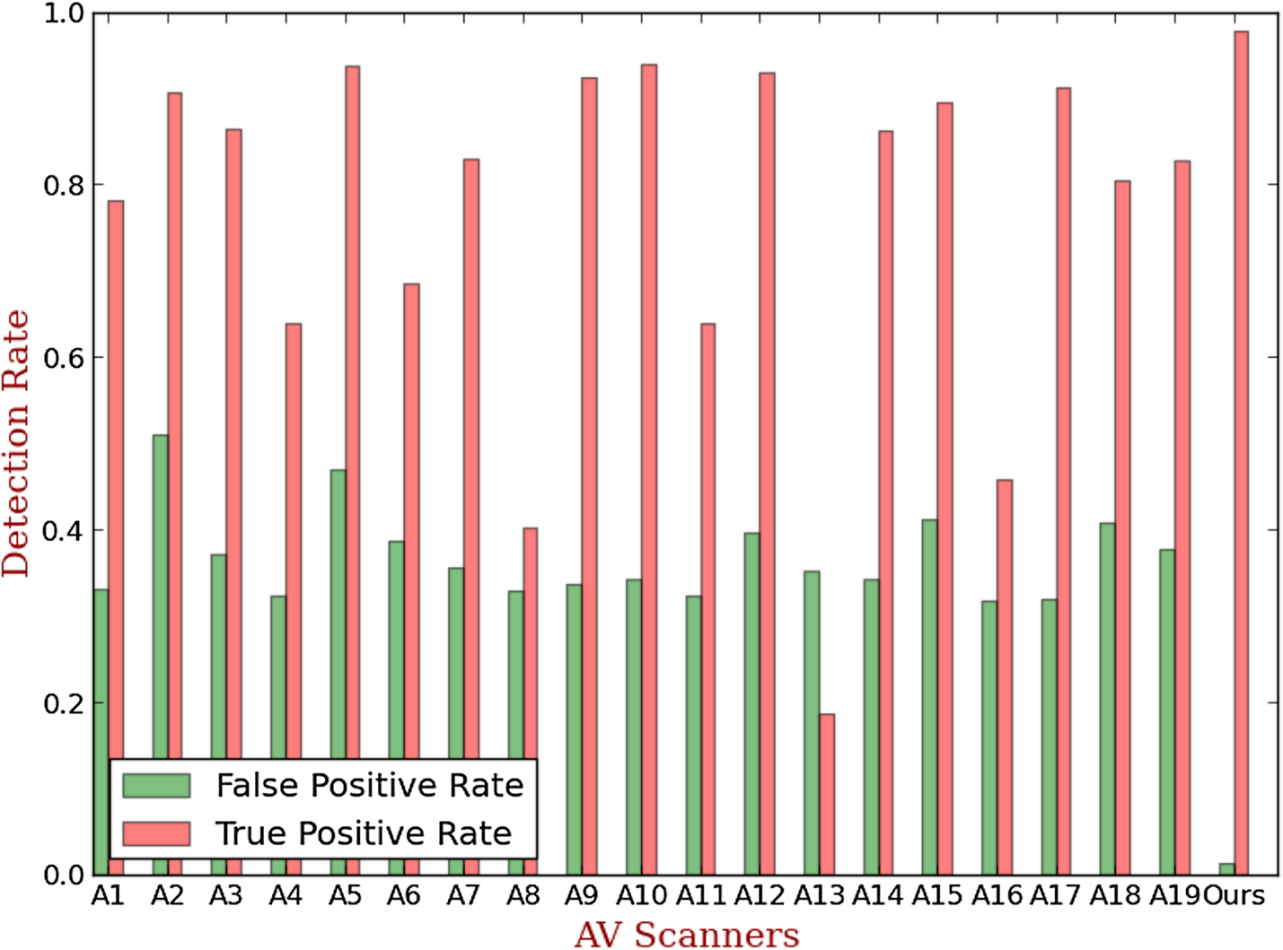
The detection performance of AV scanners and ours

Although our anomaly detection engine anomaly detection engine shows a better performance compared to related approaches, in the end it has to compete with common anti-virus products in practice. Consequently, we also compare our anomaly detection engine against the nineteen selected common AV scanners on our malware datasets. The detection rate of each scanner is computed. The nineteen AV scanners include CAT-QuickHeal, Alibaba, Symantec, ESET-NOD32, TrendMicro-HouseCall, Kaspersky, Tencent, Fortinet, Microsoft, Qihoo-360, Ikarus, Baidu, etc. These ninetees AV scanners are represented as A1-19. The detection performance of AV scanners and ours are shown in Fig. 6.

### Signature detection performance

In this experiment, we evaluate the signature classification performance of our signature detection engine based on Linear SVC–L1, Linear SVC–L2 and LinearSVM classifier, respectively.

**Fig. 7:**
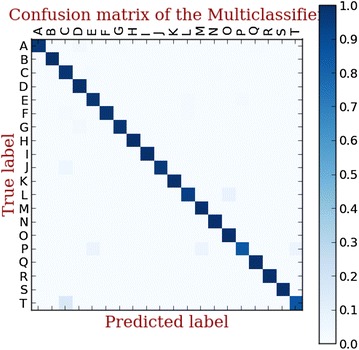
Linear SVC-L1 based multi-family classifie

**Fig. 8:**
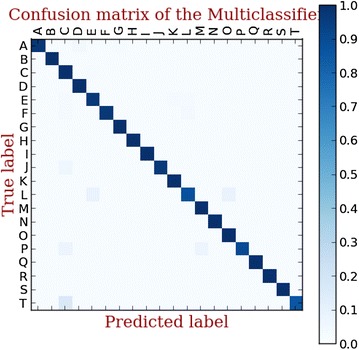
Linear SVC- L2 based multi-family classifier

**Fig. 9:**
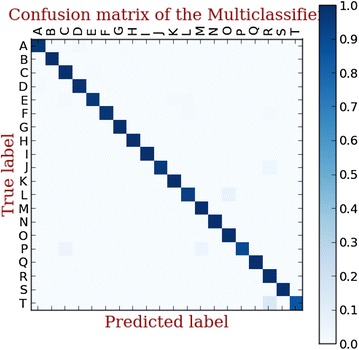
LinearSVM based multi-family classifier

We use a multi-label classification to identify the malware family of the unrecognized malicious samples. To compare the classification performance of three signature detection methods, a 10-fold validation is performed. In each fold, we split the dataset into train dataset (70 %), test dataset (30 %) respectively. The confusion matrix of classification results using three methods are shown in Figs. 7, 8, 9.

**Fig. 10:**
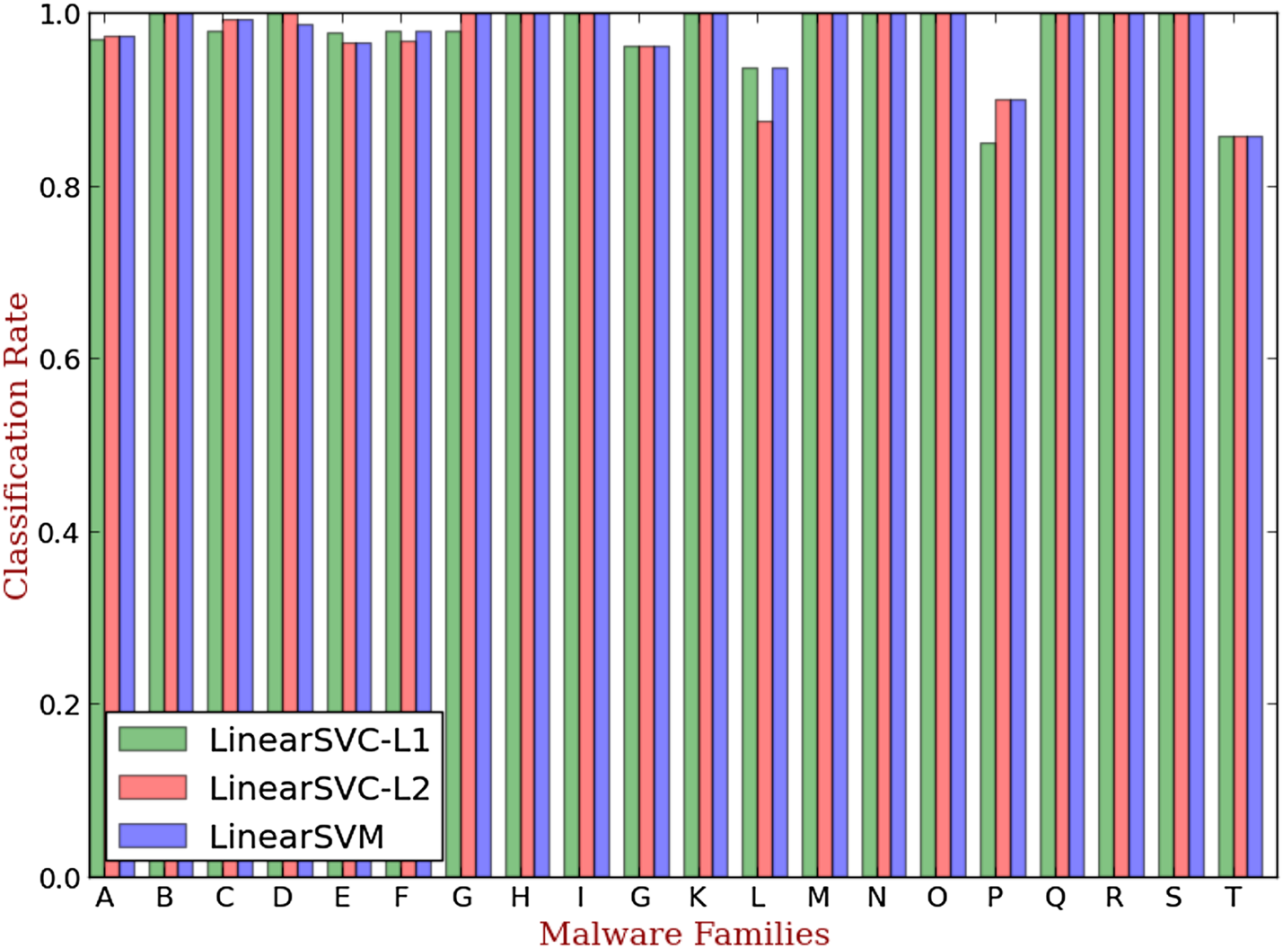
The classification performance of three methods

The classification rate of three methods is shown in Fig. 10. Above all, it is obvious that the linear SVC with L2 surpasses the other classification methods. Therefore, we use the linear SVC with L2 in our hybrid detection system.

**Fig. 11:**
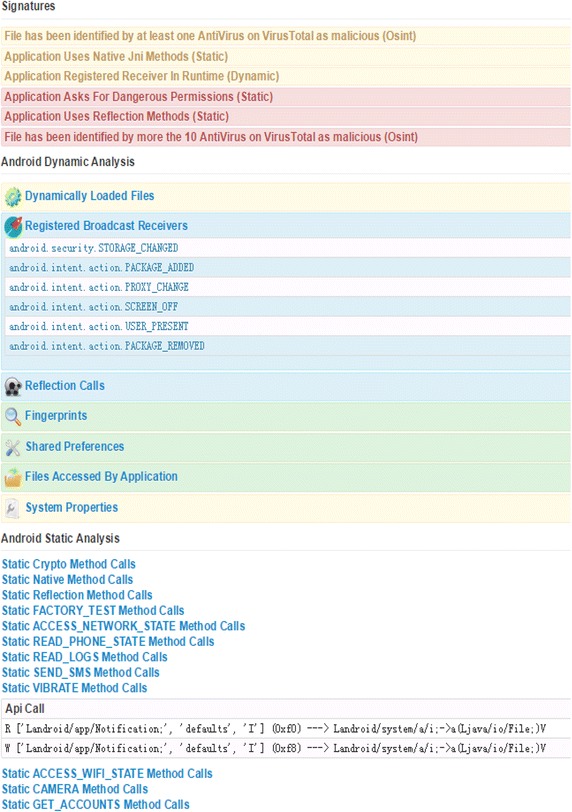
The detailed analysis report

In addition to high accuracy during misuse detection and anomaly detection, all the extracted features will be exhibited to users as a detailed analysis report. The analysis report is shown in Fig. 11. From the analysis report, an ordinary uses or expert can grasp and understand more information about the detected app.

## Discussion

In this section, we discuss the limitation of our proposed system, potential evasion techniques for our system and future work.

### Limitation 1

In our proposed system, the static analysis and dynamic analysis both work on Dalvik level. In general, our proposed system cannot handle native code or HTML5-based applications. This is because both the ARM binary running on the underlying Linux and the JavaScript code executed in WebView are not visible from a Dalvik bytecode perspective. Therefore, a future work is needed to defeat malware hidden from the Dalvik bytecode.

### Limitation 2

Although some anti-emulator detection techniques are adopted to make our dynamic analysis environment more real, more new emulator detection methods are proposed (Vidas and Christin 2014; Petsas et al. 2014; Jing et al. 2014) and can detect our emulator analysis easily. As a part of our future work, we consider adopting the dynamic hooking methods proposed in Hu and Xiao (2014) to prevent emulator evading.

### Poential evasion

As we know, learning-based detection is subject to poisoning attacks (Zhang et al. 2014). An adversary may deliberately poison the benign dataset through introducing clean apps with malicious features to confuse a training system. For example, he can inject harmless code intensively to make sensitive API calls that are rarely observed in clean apps. Once such samples are accepted as the benign samples, these APIs are therefore no longer the distinctive features to detect related malware instances.

However, our proposed system is slightly different from prior works. Firstly, the static and dynamic features are extracted to construct feature vector. Therefore, it is much harder for an attacker to make confusing samples at the behavioral-level during dynamic execution. Secondly, our anomaly detection engine serves as a sanitizer for new unknown samples. Owing to the high true positive rate of anomaly detection, any abnormal samples will be detected and further signature classification will be triggered.

### Future work

As we stated above, some future works are need to improve our proposed system.

#### Future work 1

In order to solve limitation 1, we will adopt virtual machine inspection technique (Tam et al. 2015) to include system-level events as part of the behavioral aspects (dynamic features) of an app. Incorporating system calls into our feature space can improve the behavioral models, leading to more accurate results for detecting malware using native code. Furthermore, malware utilizing root exploits can be detected and characterized more precisely by using system-level events.

To improve the code coverage in dynamic analysis, we also explore more intelligent user interactions which surpasses the MonkeyRunner (Android Developers 2015) we currently used.

#### Future work 2

As the limitation 2 stated above, in order to prevent malware from detecting emualtor and evading analysis, we will adopt new dynamic hooking mehtods (Hu and Xiao 2014) to construct a more real dynamic analysis environment.

#### Future work 3

Although we mentioned that our proposed system can be deployed in the Cloud, how to deploy it is not clear. As a important part of our future work, we will refer some recent moile cloud computing researches (Titze 2012; Chun and Maniatis 2009; Kosta et al. 2013; Borcea et al. 2015; Barakat et al. 2014) to provide a security, efficient and convient service for both app markets and ordinary users.

## Conclusion

In this paper, we proposed a novel hybrid mobile malware detection and classification system using an open-source framework CuckooDroid. Our proposed detection system integrates anomaly detection and misuse detection through static analysis and dynamic analysis of apps, providing a detail and accurate detection results. To detect zero-day malware, we design an anomaly detection engine, which exhibits amazing detection accuracy in experiment. To classify unknown malware sample, we design a signature detection engine, which can classify malware with a high positive rate. We evaluate our detection system using 6000 benign samples and 5560 malware samples. Experiment shows that our anomaly detection engine can detect abnormal malware with a high true positive rate 98.84 %, relatively low false negative rate (1.16 %) and false positive rate (1.3 %); our signature detection engine can classify 98.94 % malware samples.

Aiding by automate and comprehensive features of CuckooDroid, our detection system can be deployed in Cloud service to provide malware detection and classification for both some app markets and ordinary users through a web interface.

## References

[CR1] Aafer Y, Du W, Yin H (2013) DroidAPIMiner: mining API-Level Features for Robust Malware Detection in Android. In: International Conference on Security and Privacy in Communication Networks (SecureComm)

[CR2] Afonso VM, de Amorim MF, Gregio, Junquera, de Geus (2014) Identifying android malware using dynamically obtained features. J Comp Virol Hacking Tech

[CR3] Amos B, Turner HA, White J (2013) Applying machine learning classifiers to dynamic android malware detection at scale. In: International Conference on Wireless Communications and Mobile Computing (IWCMC)

[CR4] Android Developers (2015) Monkey Runner. http://www.developer.android.com/tools/help/ monkeyrunner_concepts.html

[CR5] Arp D, Spreitzenbarth M, Huebner M, Gascon H, Rieck K (2014) Drebin: efficient and explainable detection of android malware in your pocket. 21th Annual Network and Distributed System Security Symposium (NDSS)

[CR6] Arzt S, Rasthofer S, Fritz C, Bodden E, Bartel A, Klein J, Le Traon Y, Octeau D, McDaniel P (2014) FlowDroid: precise context, flow, field, object-sensitive and lifecycle-aware taint analysis for Android apps. In :Proceedings of the 35th Conference on Programming Language Design and Implementation (PLDI’14), pp 259–269

[CR7] Barakat OL, Hashim SJ, Abdullah RSABR, Ramli AR, Hashim F, Samsudin K, AbRahman M (2014). Malware analysis performance enhancement using cloud computing. J Comp Virol Hacking Tech.

[CR8] Borcea C, Ding X, Gehani N, Curtmola R, Khan MA, Debnath H (2015) Avatar: Mobile distributed computing in the cloud. In: Mobile Cloud Computing, Services, and Engineering (MobileCloud), 2015 3rd IEEE International Conference on March 30 2015–April 3 2015, pp 151–156

[CR9] Burguera I, Zurutuza U, Nadjm-Tehrani (2011) Crowdroid: BehaviorBased malware detection system for android. In: ACM Workshop on Security and Privacy in Smartphones and Mobile Devices (SPSM)

[CR10] Chakranomaly S, Reaves B, Traynor P, Enck W (2013) MAST: Triage for market-scale mobile malware analysis. In: ACM Conference on Security and Privacy in Wireless and Mobile Networks (WiSec). (S. Chakranomaly detection engineo)

[CR11] Chin E, Felt AP, Greenwood K, Wagner D (2011) Analyzing inter-application communication in android. In: Proceedings of the 9th Annual Symposium on Network and Distributed System Security, MobiSys 2011

[CR12] Chun BG, Maniatis P (2009) Augmented smartphone applications through clone cloud execution. In: Proceedings of the 12th conference on Hot topics in operating systems, Berkeley

[CR13] CuckooDroid 2015. http://www.cuckoo-droid.readthedocs.org/

[CR14] Enck W, Gilbert P, gon Chun B, Cox LP, Jung J, McDaniel P, Sheth A (2010) Taintdroid: An information-flow tracking system for realtime privacy monitoring on smartphones. In: Proc. of USENIX Symposium on Operating Systems Design and Implementation (OSDI), pp 393–407

[CR15] Faruki P, Bharmal A, Laxmi V, Ganmoor V, Gaur MS, Conti M, Rajarajan M (2015). Android security: a survey of issues, malware penetration, and defenses. Commun Surv Tutor IEEE.

[CR16] Gilbert, P., Chun, B-G., Cox, LP, Jung J (2011) Vision: automated security validation of mobile apps at app markets. In: Proceedings of the second international workshop on Mobile cloud computing and services, MCS’11, ACM, New York, pp 21–26

[CR17] Grace M, Zhou Y, Zhang Q, Zou S, Jiang X (2012) Riskranker: scalable and accurate zero-day android malware detection. In: Proc. of International Conference on Mobile Systems, Applications, and Services (MOBISYS), pp 281–294

[CR18] Hu W, Xiao Z (2014) Guess where i am-android: detection and prevention of emulator evading on android. HitCon

[CR19] Jing Y, Zhao Z, Ahn G-J, Hu (2014) Morpheus: automatically generating heuristics to detect Android emulators. In: Proceedings of the 30th Annual Computer Security Applications Conference (ACSAC ‘14), ACM, New York, pp 216–225. doi:10.1145/2664243.2664250

[CR20] Kosta S, Perta VC, Stefa J, Hui P, Mei A (2013) Clone2clone (c2c): peer-to-peer networking of smartphones on the cloud. In: 5th USENIX Workshop on Hot Topics in Cloud Computing (HotCloud13)

[CR21] Lindorfer M, Neugschwandtner M, Weichselbaum L, Fratantonio Y, van der Veen V, Platzer C (2014) Andrubis—1,000,000 Apps Later: a view on current android malware behaviors. In: International Workshop on Building Analysis Datasets and Gathering Experience Returns for Security (BADGERS)

[CR22] Lindorfer M, Neugschwandtner M, Platzer C (2015) MARVIN: efficient and comprehensive mobile app classification through static and dynamic analysis[J]. http://www.iseclab.org/papers/marvin_compsac15.pdf

[CR23] Petsas T, Voyatzis G, Athanasopoulos E, Polychronakis M, Ioannidis S (2014) Rage against the virtual machine: hindering dynamic analysis of android malware, In: Seventh European Workshop on System Security, pp 1–6. doi:10.1145/2592791.2592796

[CR24] Poeplau S, Fratantonio Y, Bianchi A, Kruegel C, Vigna G (2014) Execute this! Analyzing unsafeand malicious dynamic code loading in Android applications. In: Proceedings of the 21st Network and Distributed System Security Symposium (NDSS’14)

[CR25] Protalinski E (2012) A first: hacked sites with android drive-by download malware. http://www.zdnet.com/blog/security/a-first-hacked-sites-withandroid-drive-by-download-malware/11810

[CR26] Rastogi V, Chen Y, Enck W (2013) Appsplayground: automatic security analysis of smartphone applications. In: third ACM conference on Data and application security and privacy, pp 209–220. doi:10.1145/2435349.2435379

[CR27] Robotium (2014) Robotium, the world’s leading AndroidTM test automation framework. https://www.code.google.com/p/robotium/

[CR28] Sahs J, Khan L (2012) A machine learning approach to android malware detection. In: European Intelligence and Security Informatics Conference (EISIC). IEEE, pp 141e7. http://dx.doi.org/10.1109/EISIC

[CR29] Sasnauskas R, Regehr J (2014) Intent fuzzer: crafting intents of death. doi:10.1145/2632168.2632169

[CR30] Shang Y, Luo W, Xu S (2011). L-hop percolation on networks with arbitrary degree distributions and its applications. Phys Rev E.

[CR31] Spreitzenbarth M, Echtler F, Schreck T, Freling FC, Hoffmann J (2013) MobileSandbox: looking deeper into android applications. In: 28th International ACM Symposium on Applied Computing (SAC)

[CR32] Šrndić N, Laskov P (2014) Practical evasion of a learning-based classifier: a case study. In: IEEE Symposium on Security and Privacy (S&P)

[CR33] Suarez-Tangil G, Tapiador JE, Peris-Lopez P, Ribagorda A (2013). Evolution, detection and analysis of malware for smart devices. IEEE Commun Surv Tutor.

[CR34] Sufatrio, Tan DJJ, Chua T-W, Vrizlynn LL (2015). Securing android: a survey, taxonomy, and challenges. ACM Comput. Surv.

[CR35] Tam K, Khan SJ, Fattori A, Cavallaro L (2015) CopperDroid: automatic reconstruction of android malware behaviors. In: Proceedings of the Network and Distributed System Security Symposium (NDSS’15), San Diego .Internet Society

[CR36] Titze D (2012) A cloud-based security service for smartphones. Master’s thesis, Technische Universitat München

[CR37] Vidas T, Christin N (2014) Evading android runtime analysis via sandbox detection. In: Proceedings of the 9th ACM Symposium on Information, Computer and Communications Security, ASIA CCS’14, ACM, New York, pp 447–458. doi:10.1145/2590296.2590325

[CR38] Wu W-C, Hung S-H (2014) DroidDolphin: a dynamic android malware detection framework using big data and machine learning. In: Conference on Research in Adaptive and Convergent Systems (RACS)

[CR39] Yan L-K, Yin H (2012) Droidscope: seamlessly reconstructing os and dalvik semantic views for dynamic android malware analysis. In: Proc. of USENIX Security Symposium

[CR40] Zhang Y, Yang M, Xu B, Yang Z, Gu G, Ning P, Wang XS, Zang B (2013b) Vetting undesirable behaviors in Android apps with permission use analysis. In :Proceedings of the 20th ACM Conference on Computer and Communications Security (CCS’13), pp 611–622

[CR41] Zhang M, Duan Y, Yin H, et al (2014) Semantics-aware android malware classification using weighted contextual API dependency graphs[C]. Proceedings of the 2014 ACM SIGSAC Conference on Computer and Communications Security. ACM, pp 1105–1116

[CR42] Zhauniarovich Y, Ahmad M, Gadyatskaya O, Crispo B, Massacci F (2015) StaDynA: addressing the problem of dynamic code updates in the security analysis of android applications. In: ACM Conference on Data and Application Security and Privacy (CODASPY)

[CR43] Zhou Y, Jiang X (2012) Dissecting android malware: Characterization and evolution. In: Proc. of IEEE Symposium on Security and Privacy, pp 95–109

[CR44] Zhou Y, Wang Z, Zhou W, Jiang X (2012) Hey, you, get off of my market: Detecting malicious apps in official and alternative android markets. In: Proc. of Network and Distributed System Security Symposium (NDSS)

[CR45] Zhu H, Xiong H, Ge Y, Chen E (2014) Mobile App recommendations with security and privacy awareness. In: ACM SIGKDD International Conference on Knowledge Discovery and Data Mining (KDD), 2014

